# Properties and mechanisms of olfactory learning and memory

**DOI:** 10.3389/fnbeh.2014.00238

**Published:** 2014-07-07

**Authors:** Michelle T. Tong, Shane T. Peace, Thomas A. Cleland

**Affiliations:** ^1^Computational Physiology Lab, Department of Psychology, Cornell UniversityIthaca, NY, USA; ^2^Computational Physiology Lab, Department of Neurobiology and Behavior, Cornell UniversityIthaca, NY, USA

**Keywords:** olfactory bulb, synaptic plasticity, generalization, representational learning, behavior

## Abstract

Memories are dynamic physical phenomena with psychometric forms as well as characteristic timescales. Most of our understanding of the cellular mechanisms underlying the neurophysiology of memory, however, derives from one-trial learning paradigms that, while powerful, do not fully embody the gradual, representational, and statistical aspects of cumulative learning. The early olfactory system—particularly olfactory bulb—comprises a reasonably well-understood and experimentally accessible neuronal network with intrinsic plasticity that underlies both one-trial (adult aversive, neonatal) and cumulative (adult appetitive) odor learning. These olfactory circuits employ many of the same molecular and structural mechanisms of memory as, for example, hippocampal circuits following inhibitory avoidance conditioning, but the temporal sequences of post-conditioning molecular events are likely to differ owing to the need to incorporate new information from ongoing learning events into the evolving memory trace. Moreover, the shapes of acquired odor representations, and their gradual transformation over the course of cumulative learning, also can be directly measured, adding an additional representational dimension to the traditional metrics of memory strength and persistence. In this review, we describe some established molecular and structural mechanisms of memory with a focus on the timecourses of post-conditioning molecular processes. We describe the properties of odor learning intrinsic to the olfactory bulb and review the utility of the olfactory system of adult rodents as a memory system in which to study the cellular mechanisms of cumulative learning.

## 1. Introduction

**Figure 1:**
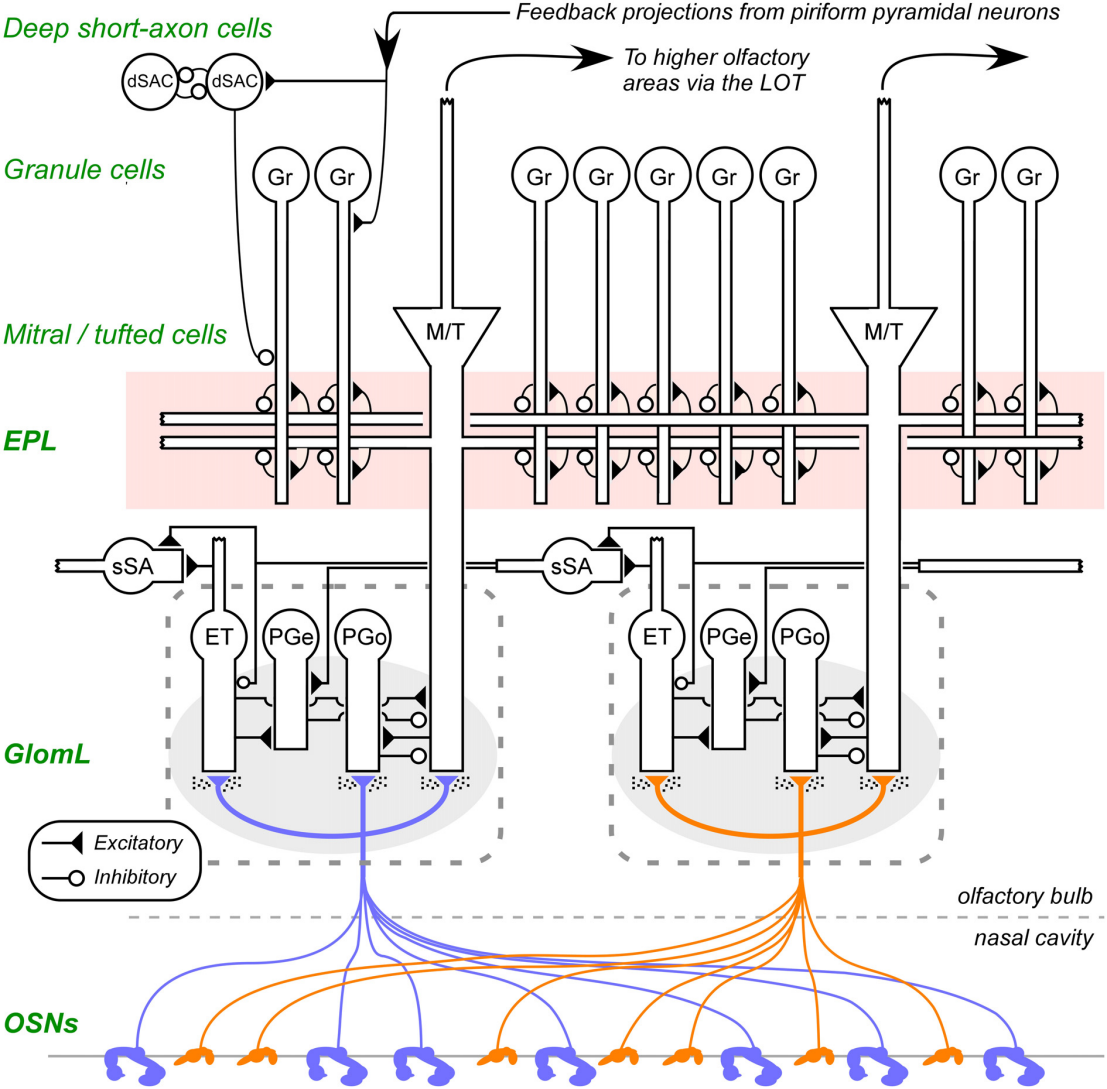
**Circuit diagram of the mammalian olfactory bulb**. The axons of olfactory sensory neurons (OSNs) expressing the same odorant receptor type converge and arborize together to form *glomeruli* (shaded ovals; two depicted) on the surface layer of the olfactory bulb. Intrinsic OB interneurons innervate each glomerulus, including olfactory nerve driven periglomerular cells (PGo), external tufted cell-driven periglomerular cells (PGe), and external tufted cells (ET). Superficial short-axon cells (sSA), closely related to PG cells and possibly part of the same heterogeneous population, are not associated with specific glomeruli but project broadly and laterally within the deep glomerular layer. Principal neurons include mitral cells and tufted cells (collectively depicted as M/T), which interact via reciprocal connections in the external plexiform layer (EPL) with the dendrites of inhibitory granule cells (Gr), thereby receiving recurrent and lateral inhibition, and project out of the OB to several regions of the brain. The heterogeneous deep short-axon cell population (dSAC) includes cells that deliver GABAergic inhibition onto granule cells and one another, and, along with granule cells, receive centrifugal cortical input from piriform pyramidal cells. OE, olfactory epithelium (in the nasal cavity); GL, glomerular layer; EPL, external plexiform layer; MCL, mitral cell layer; IPL, internal plexiform layer; GCL, granule cell layer. Filled triangles denote excitatory synapses; open circles denote inhibitory synapses. Speckles surrounding OSN terminals denote volume-released GABA and dopamine approaching presynaptic GABA_B_ and dopamine D2 receptors. Note that sSA-PG and sSA-sSA synapses are depicted as excitatory despite being GABAergic (discussed in Cleland, 2014). Figure adapted from Cleland et al. (2012).

Odor learning, like all learning, is distributed across multiple regions of the brain. Studies of learning within associative brain regions such as the hippocampus and prefrontal cortex—particularly in rodents—can utilize and manipulate olfactory stimuli just as they do other forms of sensory input (Eichenbaum et al., 1996; Eichenbaum, 1998; Law and Smith, 2012; Peters et al., 2013). Importantly, however, a substantial component of odor learning is intrinsic to the olfactory bulb (OB), and to its interactions with the piriform (olfactory) cortex to which OB mitral cells (second-order sensory neurons of the OB) project (Figure 1). Within OB proper, several lines of evidence, including N-methyl-D-aspartate (NMDA)-based synaptic plasticity (Wilson, 1995; McNamara et al., 2008), the long-term potentiation of ascending piriform pyramidal projections onto OB granule cells (Gao and Strowbridge, 2009) and odor memory persistence linked to the selective retention of adult-born interneurons in the OB (Moreno et al., 2009; Kermen et al., 2010; Sultan et al., 2010) indicate that the OB itself supports sophisticated intrinsic plasticity mechanisms that regulate the transformation of olfactory signals across the first principal sensory synapse.

The elucidation of these intrinsic learning mechanisms within OB presents both theoretical and practical opportunities. While the OB is highly interconnected with multiple cortical and subcortical regions, it is morphologically isolated. This facilitates, for example, the specific delivery of neurochemicals or virally-packaged transgenes to the OB via cannulation. The neural circuitry of the OB and the physiology of its diverse neurons are reasonably well-described (Figure 1), enabling the development of biophysically realistic models of OB function that can associate specific cellular properties and mechanisms with systems-level function and performance (Migliore and Shepherd, 2002; Li and Cleland, 2013). Specific odor-dependent behavioral paradigms have been developed that are strongly sensitive to OB manipulations and are likely to depend on OB intrinsic learning, enabling some segregation of OB-specific learning from odor learning dependent on other brain regions (Wilson and Linster, 2008). As the direct target of primary sensory neurons, the OB responds to, and differentiates among, the physical stimulus representations of odorants, but also closely apposes these bottom-up inputs with powerful top-down state-dependent and neuromodulatory influences. The olfactory system thus provides a powerful model to study *representational learning* (Bieszczad and Weinberger, 2010)—that is, the integrated effects of learning on the *forms* (or shapes) of neural representations as well as their persistence (see section 2.1). However, the OB remains underdeveloped as a model system for the neuroscientific study of learning and memory circuits. Critical elements such as the factors influencing memory persistence, the mechanistic differences between associative and nonassociative conditioning, and the signature molecular mechanisms of cellular and synaptic learning have been observed in OB but require further exploration. We here outline the features of OB-dependent intrinsic learning and review work on the structural and molecular mechanisms of memory formation, with a focus on the timecourses of learning-initiated signaling cascades and the roles of extracellular signals such as classical neuromodulators and the peptide brain-derived neurotrophic factor (BDNF). In particular, we discuss how research into representational, appetitive and cumulative learning mediated by plasticity in the olfactory system can most productively contribute to a broad understanding of general learning and memory mechanisms.

## 2. Learning in the olfactory bulb

### 2.1. Odor learning is representational

**Figure 2:**
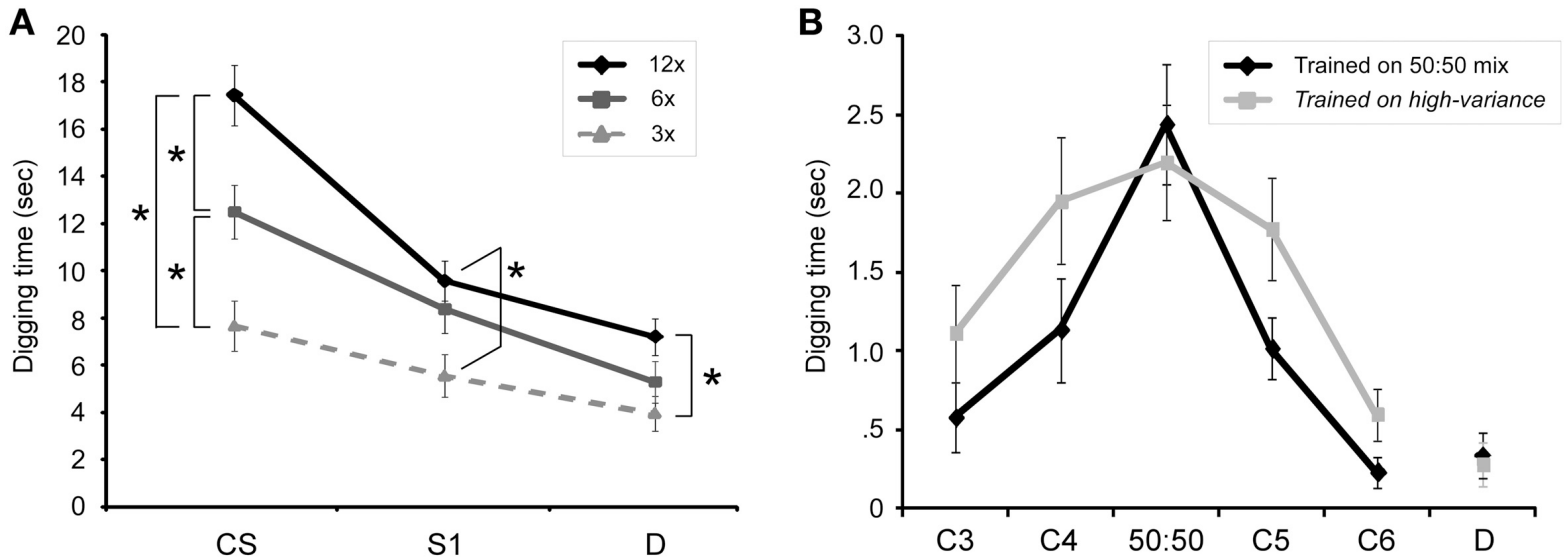
**Olfactory generalization gradients in mice**. **(A)** Mice received either 3, 6, or 12 pairings of an odor CS with buried reward, after which their perseverance in digging following randomized presentation of the odor CS, a similar odorant (S1) and a dissimilar odorant (D) was measured. Increasing the number of training trials prior to testing progressively increased perseverance and sharpened associative generalization gradients. 3x: three training trials; 6x: six training trials; 12x: 12 training trials. Asterisks denote significant pairwise differences. Figure adapted from Cleland et al. (2009). **(B)** Generalization gradients adapt to the variance of the conditioning odor. Mice received 12 pairings of an odor CS with buried reward; this CS was either a 50:50 mixture of odorants C4 and C5 (*Trained on 50:50 mix* group) or was a variable mixture of the same two odorants, varying from 95:5 to 5:95 on different trials (*Trained on high variance* group). The high-variance training group generalized significantly more broadly than the low-variance training group. Abscissa comprises a sequentially-similar homologous series of different odorants (C3, C4, C5, C6) and a dissimilar odorant (D). Figure adapted from Cleland et al. (2012).

*Learning* alters the transformation of information by a neural circuit, and *memory* refers to the persistence of that altered transformation function over time. In olfactory representational learning, the *forms* of odor representations are sensitive to learning and can be measured using behavioral generalization gradients (Cleland et al., 2002, 2009; Fernandez et al., 2009). Olfactory generalization gradients define the range of variance in odor quality that an animal will respond to as representative of a given odor, and reflect the statistical reliability of odor features (Wright and Smith, 2004). The area under the gradient, or *consequential region* (Shepard, 1987), describes the degree of certainty expressed by the animal that a stimulus of a given quality is likely to represent that learned odor or its implications. Increased pairings of odor with reward progressively sharpen the generalization gradient (Figure 2A), and manipulations of other training parameters indicate that factors that increase classical learning also increase the rate of sharpening of olfactory generalization gradients (Cleland et al., 2009). If the odor being paired with reward is itself variable in quality, however, it becomes clear that the generalization gradient does not sharpen *per se*, but progressively conforms to the actual environmental distribution of reward-predicting odor qualities as experienced by the animal (Cleland et al., 2012 Figure 2B). That is, the learning-dependent regulation of generalization gradients describes a statistical learning process by which an animal's internal odor representations become gradually and probabilistically categorical (Tenenbaum and Griffiths, 2001), evolving to correctly reflect the meaningful categories of the external olfactory environment (Cleland et al., 2012). This aspect of odor learning has been hypothesized to rely on OB circuitry both for theoretical reasons and based on results from the experimental manipulation of OB circuit function (Mandairon et al., 2006; Guérin et al., 2008; McNamara et al., 2008; Linster and Cleland, 2010; Devore and Linster, 2012; de Almeida et al., 2013; Dillon et al., 2013). Hence, in contrast to *odorants*—which are chemical stimuli, whether simple or complex—*odors* here are psychometrically defined as probability density functions of odorant quality that the animal has learned imply the same consequences, embedded within a high-dimensional similarity space that is best defined by odorant receptor activation levels (Cleland, 2008, 2014). Behaviorally-measured generalization gradients constitute one-dimensional trajectories within this high-dimensional space, essentially estimating the changing form of the odor representation via sampling. The key point is that memory content is not a constant, but changes with learning and over time just as memory strength and persistence do. By providing quantifiable, interpretable measures of memory content as it evolves, odor generalization gradients illustrate the advantages of representational learning systems for the study of learning and memory mechanisms.

### 2.2. Appetitive odor learning is cumulative and incremental

**Figure 3:**
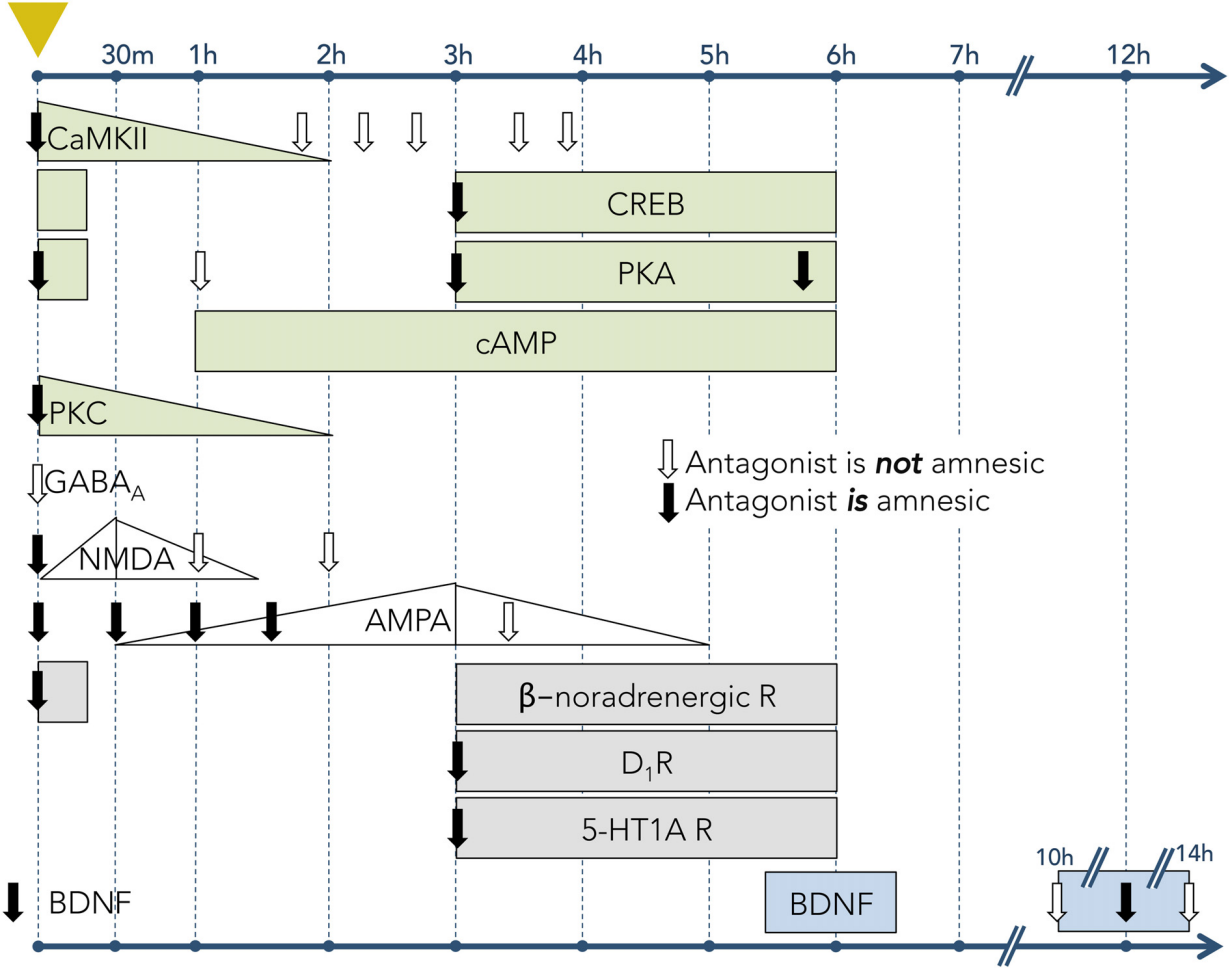
**Timecourses of activity for learning-associated molecular mechanisms in hippocampal tissue following one-trial inhibitory avoidance (IA) training**. Leftmost dashed vertical line (marked with yellow triangle) denotes the time of IA conditioning. Subsequent vertical lines denote timepoints following the trial. Rows correspond to particular mechanisms (signaling molecule, receptor, or neuromodulator). Downward-pointing arrows indicate timepoints when antagonists to the corresponding receptor or signaling pathway were infused into the hippocampus; solid arrows denote that the antagonists had an amnesic effect on LTM (when tested 24–48 h after conditioning); open arrows indicate no amnesic effect on LTM. Filled shapes denote the experimentally-measured expression levels of receptors or signaling molecules. Increasing or decreasing levels are depicted when known; rectangles depict measurable levels when relative abundances were not measured. Light green shapes represent intracellular signaling pathways, including Ca2+/calmodulin-dependent protein kinase II (CaMKII), cAMP response element-binding protein (CREB), protein kinase A (PKA), cyclic adenosine monophosphate (cAMP), and protein kinase C (PKC) (Data from: Jerusalinsky et al., 1994; Wolfman et al., 1994; Bernabeu et al., 1996, 1997a,b; Bevilaqua et al., 1997; Cammarota et al., 1997; Izquierdo et al., 1997). White shapes represent ionotropic receptors for neurotransmitters, including γ-Aminobutyric acid A (GABA_A_) receptors, N-methyl-D-aspartate (NMDA) receptors, and α-amino-3-hydroxy-5-methyl-4-isoxazolepropionic acid (AMPA) receptors (Data from: Izquierdo et al., 1992; Jerusalinsky et al., 1992; Bernabeu et al., 1997c). Gray shapes represent the activity of receptors for neuromodulators, including β-noradrenergic receptors, dopamine type 1 receptors (D_1_R), and serotonin 1A receptors (5-HT1A) (Data from: Izquierdo et al., 1992; Bernabeu et al., 1996; Bevilaqua et al., 1997). Blue shapes represent brain-derived neurotrophic factor (BDNF; Data from Bekinschtein et al., 2007).

The cellular and synaptic neurophysiology of mammalian learning and memory is substantially based on fear conditioning. The advantage of the conditioned fear model is that strong, discrete, and easily measurable memories can be generated by single learning trials, avoiding the complexity and additional questions imposed by the need to integrate the cumulative effects of multiple learning events. The persistence of these memories is a function of the unconditioned stimulus amplitude—e.g., footshock current—but commonly extends to several days (Bekinschtein et al., 2007), enabling study of the sequential transitions in their structural, biochemical, and molecular substrates that occur over time. The clearest example of these gradually transforming dependencies is the protein synthesis requirement for long-term (many hours to days or more) but not short-term (up to a few hours) memory (Davis and Squire, 1984; DeZazzo and Tully, 1995), though additional phases of memory have been defined in some systems. Moreover, many specific cellular signaling cascades, induced by fear conditioning events and underlying the relevant learning, have been described; whereas most of these processes are initiated immediately after the causal event, several have been described that are initiated minutes or even hours later (Figure 3).

Olfactory appetitive learning, in contrast, is gradual, cumulative, and statistical (Cleland et al., 2009, 2012 Figure 2). The richness of the OB learning model that is gained by its statistical and representational character also imposes a cost in terms of unavoidable complexity. For example, the distribution of repeated training events in time will always be a factor; *massed* versus *spaced* learning schedules are well known to affect memory persistence (Tsao, 1948; Menzel et al., 2001; Kermen et al., 2010), and intertrial interval timing can even determine which areas of the brain are most immediately responsible for nonassociative odor learning (McNamara et al., 2008). This complexity, however, is manageable, and is substantially mitigated by the theoretical tractability and experimental accessibility of the OB as well as the elucidation of plasticity-related molecular cascades in other cortical memory systems. Studies of representational plasticity and memory processes in primary and secondary sensory cortices offer a singular opportunity to understand a new dimension of memory—plasticity in form as well as persistence—in preparations within which its form can be measured physiologically as well as psychophysically (Bieszczad and Weinberger, 2010). This is critical for a mechanistic understanding of statistical learning, in which repeated stimulus experiences are not identical and part of the challenge of learning is to estimate the intrinsic variability of meaningful stimuli and the relationship between stimulus quality and outcome.

It is worth noting that odor learning in OB is not always gradual and cumulative. Fear conditioning to odorants induces strong one-trial learning, which appears to involve plasticity-related molecular changes in the OB (Jones et al., 2007). However, this aversive learning appears to substantially *broaden* olfactory generalization, rather than sharpen it as is observed in appetitive odor learning (Chen et al., 2011). This can be interpreted as adaptive, in that broad generalization is the safe, conservative response to a dangerous odor of uncertain variance, but it raises important mechanistic questions: what would be the cumulative effect of multiple aversive conditioning trials, for example, or how would this broad generalization gradient interact with strong, preexisting, non-aversive odor representations? It also suggests that the mechanisms underlying the effects of aversive conditioning in OB may differ qualitatively from those underlying appetitive conditioning at some level of organization.

Odor learning in neonates also is qualitatively different from that described in adults. First, neonatal odor learning is more heavily OB-dependent than it is in adults, in part because downstream learning areas such as the amygdala are not yet functional (Berdel et al., 1997; Sullivan, 2003). Second, neonatal odor learning is substantially stronger and less conditional than it is in adults, exhibiting a nearly all-or-none quality that contributes to the rapid learning of maternal and nest odors (Sullivan et al., 1991, 2000). Accordingly, studies of the molecular cascades and mechanisms underlying intrinsic OB learning are most advanced in neonates (McLean and Harley, 2004; McLean et al., 2005; Grimes et al., 2012; Lethbridge et al., 2012). Many of these mechanisms, however, are likely to be conserved in some form to underlie the incremental appetitive learning exhibited by adults. For example, norepinephrine (NE) and the intracellular cascades that it initiates play a central role in neonatal olfactory learning (Sullivan et al., 1989; Yuan et al., 2003). Indeed, the innate hyperresponsivity of the neonatal locus coeruleus (LC) is the underlying mechanism of one-trial odor learning in neonates (Sullivan, 2001, 2003; Moriceau and Sullivan, 2005), and NE delivery to the OB is sufficient to induce odor preference learning in neonates (see section 4.1). In adults, in contrast, LC responsivity is much more measured and conditional. Nevertheless, NE in the adult OB is essential for even the basic nonassociative learning processes underlying odor habituation (Guérin et al., 2008; Shea et al., 2008; Moreno et al., 2012), and selective NE receptor antagonists infused into OB impair conditioned odor preference learning, recognition memory, and near-threshold odor identification (Guérin et al., 2008; Escanilla et al., 2010, 2012; Linster et al., 2011; Manella et al., 2013). In this way, the cellular mechanisms of memory elaborated by one-trial learning paradigms can serve as the basis for study of the more complex problem of ongoing appetitive learning.

### 2.3. Advantages of olfactory bulb learning models

**Figure 4:**
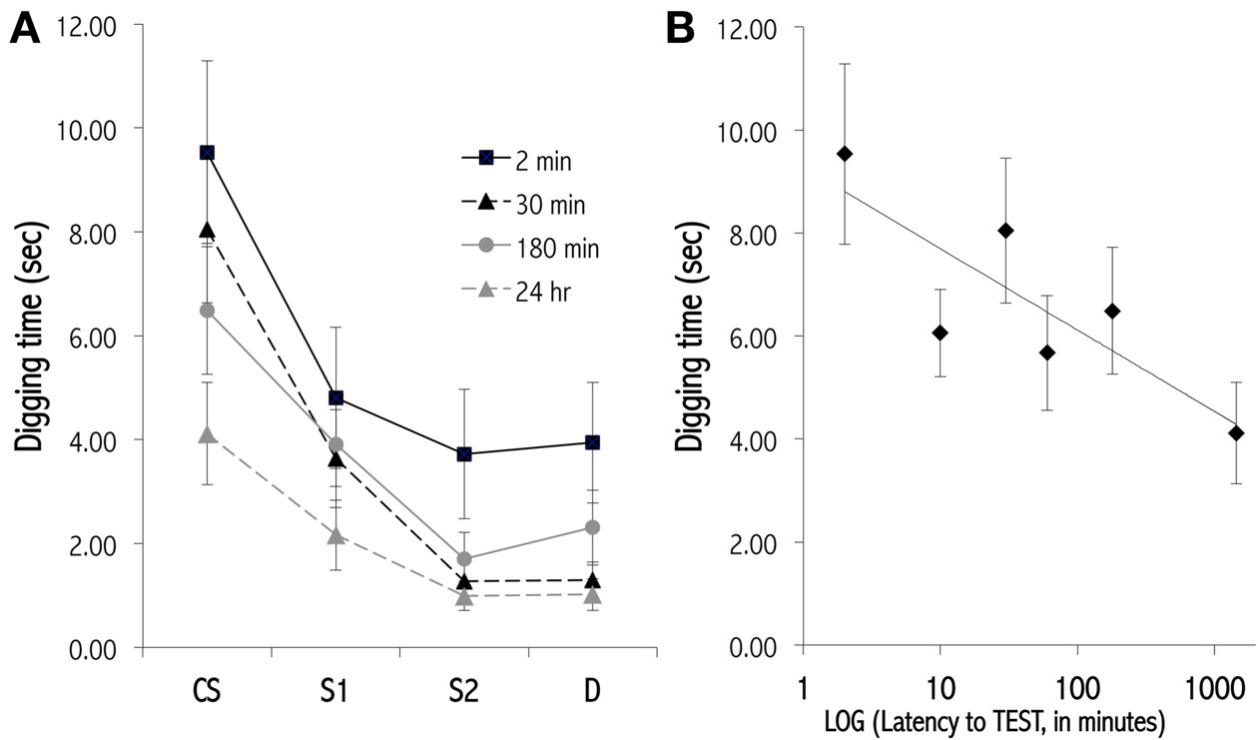
**Olfactory generalization gradients measured at various latencies after conditioning**. **(A)** Progressive decay of a newly learned olfactory generalization gradient over an STM timescale. Mice received 12 massed training trials in which they dug in a dish of sand scented with a 1.0 Pa conditioned odor to retrieve a food reward. Separate cohorts of conditioned mice then were tested at different latencies for their perseverative responses (digging times) to the odor CS, a highly similar odorant S1, a moderately similar odorant S2, and a structurally and perceptually different odorant D. Responses declined and generalization gradients flattened with greater training-testing latencies. Methodology follows that of Cleland et al. (2009). Four of six latencies tested are depicted for clarity. **(B)** Lin-log plot of digging time in the CS during testing at all six latencies tested (2, 10, 30, 60, 180, 1440 min). Data are fit with the regression line *y* = −0.687ln(x) + 9.278, *R*^2^ = 0.683.

**Figure 5:**
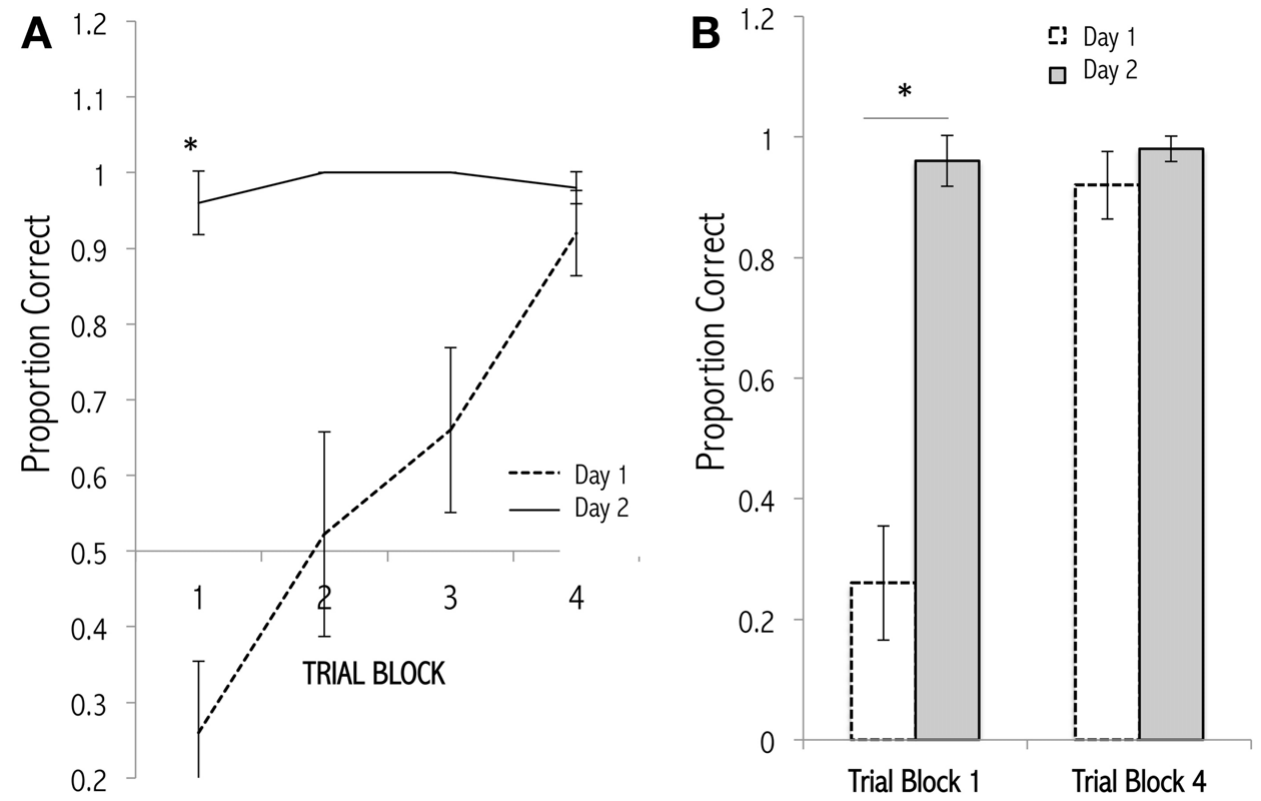
**Learning curves for an odor discrimination task**. **(A)** Mice received 20 trials of discrimination training (Cleland et al., 2002) in which they learned to choose a rewarded conditioned odor (1.0 Pa) over a distractor odor (Day 1). Twenty-four hours later, the discrimination training was repeated (Day 2). The correct trials were scored and averaged across animals. Trials are grouped into 4 blocks of 5 consecutive trials for display and analysis. A steady improvement across trials on Day 1 is remembered 1 day later. **(B)** Data from trial blocks 1 and 4 replotted for comparison. Comparing trial block 1 (trials 1–5) between days, mice performed significantly better on the second training session, indicating a robust retention of odor memory [*asterisk*: *t*(8) = 7.5593, *p* < 0.001]; no other comparisons approach statistical significance. Comparable findings have been observed by Schellinck et al. (2001).

The OB provides both practical and theoretical advantages for study of the molecular and structural mechanisms involved in memory. Practically, pharmacological agents can be infused selectively and locally into the OB. Intrinsic OB circuits display functional plasticity similar to other regions of the brain, including long-term synaptic potentiation (Gao and Strowbridge, 2009) and adult neurogenesis (Lledo et al., 2006), and are reconfigured substantially by neuromodulatory inputs (Devore and Linster, 2012). Established behavioral paradigms enable insight into the changing form as well as the persistence of odor representations over time, and physiological studies enable measurements of direct correspondence between environmental changes, behavioral performance, and the synaptic and molecular changes that occur in neural circuitry (Abraham et al., 2012, 2014; Qiu et al., 2014). In particular, odor learning exhibits varying memory durations that are related to behavioral task parameters and depend on evolving physiological substrates for short-term memory (Figure 4; McNamara et al., 2008), intermediate-term memory (Grimes et al., 2011), and long-term memory (Figure 5; Lazarini and Lledo, 2011).

#### 2.3.1. Habituation and cross-habituation

In this non-associative olfactory learning paradigm, animals first are habituated to an odorant, responding to repeated presentations with progressively lower investigation times. Some time after habituation, they are presented again with that odorant, or with a series of structurally and perceptually similar odorants (*cross-habituation*, also referred to as spontaneous discrimination). Perceptually distinct odors elicit normal, non-habituated investigation times, but odorants similar to the habituated odorant elicit reduced, partially-habituated responses depending on the degree of similarity between the habituated and test odorants. A generalization gradient therefore can be constructed by presenting a battery of similar odorants to habituated animals and measuring the pattern of cross-habituation among odorants (Cleland et al., 2002). Interestingly, memory for odorant habituation acquired on short timescales (tens of seconds) is predominantly mediated within piriform cortex (Wilson, 2009), whereas habituation on the minutes timescale is localized within OB (McNamara et al., 2008; Chaudhury et al., 2010). Habituation and cross-habituation memory persistence is sensitive to the degree of habituation, declining over a 10–20 min period in standard protocols (Freedman et al., 2013). Both the extent and persistence of cross-habituation memory are regulated by neuromodulatory and hormonal effects in the OB as well as task parameters and state variables (Mandairon et al., 2006, 2008; Dillon et al., 2013; Manella et al., 2013).

#### 2.3.2. Associative generalization

Generalization gradients also can be measured in response to odorants that are conditioned via associative pairing with reward (Cleland et al., 2002, 2009). After conditioning, animals are tested with batteries of structurally and perceptually similar odorants, often in a digging task where the odorant cue signals a buried reward. The animals' perseverance, measured as time spent digging, in pursuit of an expected reward (that is not present in test trials) declines with increasing perceptual dissimilarity between the conditioned and test odorants. The breadths and forms of these gradients are sensitive to determinants of learning and to the statistical variance in odorant CS quality across conditioning trials (Cleland et al., 2009, 2012) and also are sensitive to the pharmacological and neuromodulatory manipulation of OB circuitry (Zimering and Cleland, 2011). Associative odor learning based on a standard short-term conditioning paradigm (a single series of up to twelve massed conditioning trials) progressively decays over a timescale of several hours (Figure 4), though this timescale is likely to be sensitive to training parameters.

#### 2.3.3. Odor discrimination

Odor discrimination is the most commonly used olfactory learning model, and subsumes many radically different conditioning paradigms and performance metrics. The distinguishing feature of this task is that animals are motivated to distinguish between two or more odors with different learned contingencies (e.g., one is rewarded and the other not), such that it tends to measure an animal's capacity to learn a given discrimination rather than to measure an odor representation *per se*. Automated tasks with relatively nonintuitive metrics (e.g., odor-specified left-right selection or go/no-go tasks) may utilize hundreds of training trials, whereas tasks with more intuitive (to the animal) metrics such as odor-cued digging often require less than 20 trials to reach criterion. The dependence of odor discrimination performance on OB circuitry corresponds closely with the difficulty of the discrimination (Rinberg et al., 2006), which corroborates theoretical proposals that OB circuitry serves in large part to identify which statistical differences among inputs correspond to meaningfully different odorants, and which are simply variations of a single odor that should be generalized (Cleland et al., 2012).

#### 2.3.4. Olfactory performance depends on memory

**Figure 6:**
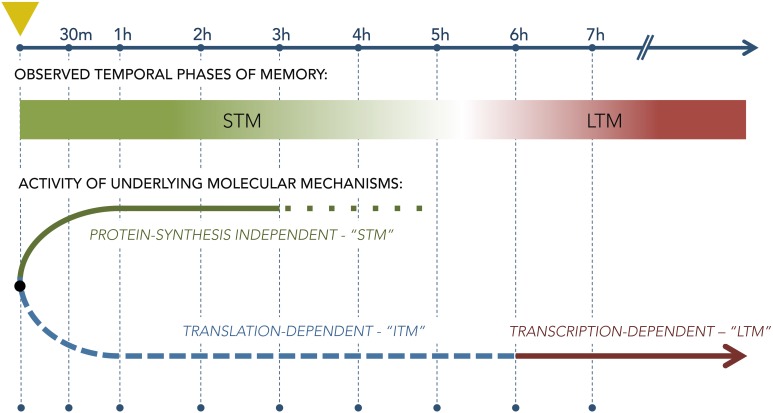
**Diagram of the separate molecular pathways underlying short-term and long-term memory as derived from studies using single-trial aversive training paradigms**. Leftmost dashed vertical line (marked with yellow triangle) denotes the the time of conditioning; subsequent vertical lines denote timepoints following the trial. The lower diagram illustrates the theoretical bifurcation of memory mechanisms into a protein synthesis-independent STM stream that governs behavior for the first few hours after training and a separate, protein synthesis-dependent LTM stream that begins to govern behavior only thereafter. The relationship, if any, between these two streams during multiple repeated conditioning trials is not clear.

In olfaction, memory does not serve only to remember odors past, but is also a critical factor in realtime perceptual processing, even within OB and piriform cortex (Wilson and Stevenson, 2003a,b; Zucco et al., 2014). Hence, short-term and long-term memory processes are likely to be highly interactive and conditional; e.g., the form of a long-term memory should acquire the evolving characteristics of accumulating short-term memory processes during multitrial odor learning tasks or natural learning scenarios. That is, though it is established in general that STM and LTM processes are initiated separately—i.e., LTM is not simply a continuation of STM (Figure 6; Izquierdo et al., 1999)—it also is true that LTM must be able to be repeatedly updated based on new information even before it is first behaviorally expressed. One likely scenario is that short-term learning and memory processes contribute to this updating—a hypothesis that the olfactory appetitive learning and memory model is well-poised to test.

## 3. Molecular and structural mechanisms of learning and memory

Memory mechanisms are heterogeneous in form, structure, and timecourse, yet exhibit many commonalities across regions of the brain. We here separate these mechanisms into two broad categories: *molecular*, which includes intracellular cascades, molecular signaling, neuromodulatory influences, activity-dependent protein synthesis, and epigenetic modifications, and *structural*, which includes physiological changes such as long-term potentiation or other synaptic weight modifications, alterations to neuronal morphology such as dendritic branching, changes to terminal shapes or numbers, and ancillary modifications such as effects on glia or cell adhesion to the extracellular matrix, as well as changes to neuron number via adult neurogenesis or selective apoptosis. Mechanisms from these categories often are interdependent, and exhibit characteristic response timecourses that underlie memory-related changes. In this section, we review selected learning models and mechanisms drawn primarily from the hippocampal literature, focusing on models with well-developed response timecourses and signaling mechanisms for which there is evidence of relevance to OB learning as well.

### 3.1. Molecular mechanisms

Inhibitory avoidance (IA) is a well-established behavioral paradigm for one-trial fear conditioning that offers a simple analog measure of memory “strength.” IA memories can persist strongly for days, enabling study of both short-term and long-term memory mechanisms. If entering a darkened chamber or stepping down from a platform results in footshock on the conditioning trial, a normal animal will hesitate, in subsequent test trials, before again entering that chamber or stepping down. The delay in seconds before again entering the chamber or stepping down is a robust measure of the strength of the action-consequence association. Much of what is known about the molecular mechanisms of memory and their timecourses in mammalian systems has been developed using this task.

#### 3.1.1. Long-term memory

IA conditioning leads to a rapid elevation in calcium/calmodulin-dependent protein kinase II (CaMKII) levels in the hippocampus. This in turn enhances the phosphorylation of cyclic AMP (cAMP) response element binding protein (CREB) (Miyamoto, 2006) and promotes the formation of complexes with ionotropic glutamate NMDA receptors (Sanhueza and Lisman, 2013), which have been shown to play a functional role in learning and memory (reviewed in Danysz et al., 1995). Blocking CaMKII activity immediately after IA training substantially reduced animals' fear responses when measured 24 h later (i.e., LTM). However, blocking CaMKII activity 30 min after IA resulted in a weaker LTM deficit, and blockade 2–4 h after IA had no effect on LTM at all (Figure 3; Wolfman et al., 1994). These findings indicate that CaMKII plays a crucial role early in the memory induction process, and that its functional role in LTM formation is confined to a specific period following learning. The neurotrophin BDNF also plays a critical signaling role in LTM induction (Figure 3). For example, blockade of BDNF signaling through its TrkB receptor, or through function-blocking anti-BDNF, disrupted LTM but not STM for a conditioned IA event, whereas infusion of recombinant BDNF into hippocampus rescued IA memory from amnesia induced by glucocorticoid receptor blockade (Chen et al., 2012).

Other studies have demonstrated the early involvement of the cAMP–protein kinase A (PKA)–CREB pathway in LTM formation. Cyclic AMP levels in the hippocampus begin to rise about 30 min following IA training, peak at 3 h after training, and decrease to baseline levels circa 6 h after training (Bernabeu et al., 1996, 1997a). PKA activity and CREB phosphorylation (pCREB levels), in contrast, both exhibit two distinct peaks: one immediately following training and another beginning roughly 3 h thereafter and persisting until 6 h, but not 9 h, post-conditioning. The second of these peaks coincides with peak hippocampal cAMP levels (Bernabeu et al., 1997a). Injection of the PKA inhibitor KT5720 into the hippocampus 0–6 h after conditioning impairs IA memory when tested 24 h after training (Bernabeu et al., 1997a). Similarly, injections of CREB antisense oligonucleotides into the amygdala impaired LTM in the IA task (Canal et al., 2008), and infusions of antisense CREB oligonucleotides into the hippocampus prior to water maze training blocked 48-h LTM while sparing 4-h STM (Guzowski and McGaugh, 1997). Mutant mice that lack the α and β isoforms of CREB also exhibit impaired LTM consolidation, but normal STM, on a contextual fear conditioning task (Bourtchuladze et al., 1994).

In a multi-trial, appetitive learning paradigm based on the radial arm maze task, increased PKA activity and CREB phosphorylation levels were observed in the hippocampus after the fourth consecutive day of training, but not after the first day, in contrast to the immediate same-day effects observed in IA studies (Mizuno et al., 2002). A similar contrast between IA conditioning and appetitive learning effects has been described with learning-associated BDNF activation. Infusions of function-blocking anti-BDNF antibody into the hippocampus prior to, but not 6 h after, IA training block LTM, indicating that BDNF activity around the time of learning sets the stage for eventual LTM consolidation (Alonso et al., 2002). In contrast, on an appetitive radial arm maze task, BDNF mRNA levels in hippocampus increased only after 8, but not 4, consecutive days of conditioning, and even then mRNA levels were significantly elevated only after 15 min, but not immediately, following training (Mizuno et al., 2002). A similar increase in BDNF mRNA levels, with a comparable 15-min delay, also was observed after 28 days of training with this task (Mizuno et al., 2000). (It remains unclear whether the levels observed at 8 and 28 days represent a continuous elevation in task-induced BDNF mRNA transcription across those days or reflect multiple peaks in BDNF mRNA activity). These findings suggest that similar molecular mechanisms can mediate multi-trial appetitive learning as underlie fear-based single-trial learning, but that the timecourses can differ. It is in these latter contingencies that the richness of appetitive learning studies is likely to contribute most significantly to general studies of learning and memory. Should LTM be modeled as a statistical evidence accumulation system, in which LTM is formed only after enough evidence has accumulated that the cue-reward association is reliable and likely to remain true over time? How is this compatible with the evidence that, in IA training, LTM induction is initiated immediately after learning (and is not dependent upon intact STM), even though it cannot govern behavioral responses until hours later? Once LTM is induced, how is the persistence of that memory governed? How does existing LTM contribute to STM formation over multi-day training sequences? What factors contribute to the timescales, selectivity, and stringency of new memory formation?

#### 3.1.2. Short-term memory

The formation and maintenance of short-term memory (STM), which are independent of protein synthesis, rely on different molecular mechanisms than those underlying LTM (Izquierdo et al., 1999). To elucidate these different mechanisms, animals were conditioned using the IA protocol, immediately infused with one of a battery of antagonists into the hippocampus, and behaviorally tested for memory retention at 1.5 h (STM) and 24 h (LTM) after training (Izquierdo, 2000). The study showed that STM formation required cyclic GMP (cGMP), mitogen-activated protein kinase kinase (MAPKK), and PKA, but did not depend on protein kinase G (PKG), protein kinase C (PKC), or CaMKII. (Note that this dependence of STM on PKA differs from the PKA-independent STM of neonatal OB as described above). Additionally, infusions of either an AMPA receptor antagonist or a γ-aminobutyric acid (GABA) subtype A receptor agonist into the entorhinal cortex prior to training impaired STM when tested at 1.5 h following training, but did not impair LTM when tested 24 h after conditioning (Izquierdo et al., 1998). Critically, these results demonstrated that LTM does not derive from STM representations, in that LTM formation does not depend on intact STM. Instead, at least two distinct cascades of events are set in motion after training (Figure 6), one of which enables rapid behavioral adjustment but decays in several hours (STM), and the other of which is longer-lasting but cannot be behaviorally expressed for the first few hours after conditioning (LTM). Interestingly, the distinct STM and LTM pathways - and many of their mechanistic elements - are common across widely divergent clades, including mollusks and insects as well as vertebrates (Davis and Squire, 1984; DeZazzo and Tully, 1995; Blum et al., 2009), suggesting that these properties have been strongly conserved.

A distinct, intermediate phase of memory, termed intermediate-term memory (ITM), also has been defined, originally in *Aplysia californica*. It is characterized primarily by its dependence on protein translation but not on transcription (Sutton et al., 2001, 2002), although a separate, mechanistically distinct form of ITM in *Aplysia* also has been described (Sutton et al., 2004). Though the timescales of these memory phases in *Aplysia* differ from their mammalian analogs, a translation-dependent, transcription-independent ITM for conditioned odor preference also has been identified in neonatal rat OB (Grimes et al., 2011). Infusion of anisomycin, a translation blocker, immediately after conditioning had no effect on odor memory when the rat pups were tested one or 3 h later, but eliminated the memory when tested 5 or 20 h after conditioning. When actinomycin, a transcription blocker, was similarly infused, memory at 1, 3, and 5 h was comparable to control animals, but an impairment of odor memory was observed at 24 h. It is likely that the mechanism underlying memory during this intermediate period (~5 h after conditioning) is simply the LTM mechanism; that is, it is in this time window that new proteins begin to be required for memory maintenance, but the translation of existing mRNA transcripts provides a sufficient supply for a limited time. This interpretation is supported by subsequent results demonstrating that PKA blockade blocks 5-h ITM and 24-h LTM, but not 3-h STM (Grimes et al., 2012).

### 3.2. Structural mechanisms

Long-term memory has long been associated with persistent structural changes in specific brain regions involved in the formation of the memory. Many of the molecular mechanisms characterized in LTM induction and maintenance also have been shown to influence these structural changes, which may in some cases be the primary effectors of the memory. We briefly review some of these structural mechanisms here.

#### 3.2.1. Long-term potentiation

Over decades of research, considerable debate has arisen about whether, and to what extent, long-term synaptic potentiation (LTP, Bliss and Lømo, 1973) underlies or otherwise corresponds to behaviorally-measured LTM (Izquierdo, 1993). The arguments in favor of their relationship were strengthened by the elucidation of two distinct forms of hippocampal long-term plasticity (LTP), a short-duration early form (E-LTP) and a longer-lasting late form (L-LTP) distinguished primarily by the latter's dependence on protein synthesis. Specifically, the persistence of LTP in the CA1 region beyond roughly 4 h depends on mRNA and protein synthesis (Frey et al., 1988; Bliss and Collingridge, 1993); translation blockers injected into rat dentate gyrus during *in vivo* LTP induction caused synaptic potentiation to decay within 3–4 h (Krug et al., 1984). This timescale closely resembles the protein-synthesis dependency of LTM observed in behavioral studies. Similarly, after LTP induction by a tetanic stimulation of afferent fibers in hippocampal slices, any further tetanus to the afferent within 3 h generates only short-term plasticity across the synapse, whereas after 4 h the same tetanus could generate a longer-lasting potentiation over and above the initially induced LTP level (Frey et al., 1995). The timescale of this effect also corresponds with the STM/LTM distinctions described above, and additionally suggests that LTM expression may free up resources needed for further learning. Finally, several molecular mechanisms associated with memory induction and persistence also regulate LTP. For example, CaMKII activity is necessary for LTP induction (Malinow et al., 1989), PKC inhibition immediately following induction leads to early decay of potentiation (Wang and Feng, 1992), and PKA inhibition prior to LTP induction limits the persistence of LTP to roughly 3 h (Frey et al., 1993). BDNF also facilitates the induction of LTP in hippocampal slices (Korte et al., 1995), and the application of BDNF in the presence of protein synthesis inhibitors is sufficient to transform a short-lasting LTP to a longer-lasting form (Lu et al., 2008), suggesting a role for BDNF in the determination of long-term functional plasticity that is comparable to its necessary and sufficient role in determining LTM persistence (Bekinschtein et al., 2008). Moreover, blockade of BDNF signaling immediately following LTP induction reduced LTP persistence. Specifically, LTP induction in slices generated a transient peak in the phosphorylated form of the TrkB receptor for BDNF; pTrkB levels rose 15 min following induction, peaked at 30 min, and slowly declined to baseline over 2 h (Lu et al., 2011). Preventing TrkB activation with TrkB-IgG at the 30-min peak, but not at 60 min post-induction, inhibited persistent LTP (Kang et al., 1997). The timecourses of these interactions also correspond to those of the early biochemical cascades involved in LTM formation as discussed above.

#### 3.2.2. Neuronal and synaptic morphology

Changes in neuronal morphology, such as the growth of new dendritic spines, have been shown to accompany novel experiences (Leggio et al., 2005; Jung and Herms, 2014). Importantly, the stabilization of new dendritic spines underlies at least some LTMs (Yang et al., 2009), indicating that durable modifications of the synaptic weights within neuronal networks mediated by physical spines is a structural mechanism underlying memory persistence (reviewed in Ramiro-Cortés et al., 2014; Sotelo and Dusart, 2014). The specific roles of these morphological elements are further emphasized by the dependence of LTM on intact cytoskeletal dynamics (Lamprecht, 2014). Notably, BDNF and other neurotrophins associated with memory regulation have been strongly implicated in the modification and maintenance of both synaptic efficacy and dendritic morphology (reviewed in McAllister et al., 1995; Castello et al., 2014; Zagrebelsky and Korte, 2014).

#### 3.2.3. Adult neurogenesis

Learning and memory in the hippocampus and olfactory bulb also are associated with the incorporation of new adult-born neurons. The proliferation of new neurons ceases prior to adulthood in most brain regions, with the exception of the hippocampus and OB, and possibly the hypothalamus (Cheng, 2013). Hippocampal progenitor cells are produced in the subgranular zone (SGZ) of the hippocampus and migrate a short distance to the granule cell layer of the dentate gyrus (DG); in contrast, OB progenitor cells are produced in the subventricular zone (SVZ) and migrate to the OB along the rostral migratory stream for 10–14 days before arriving in the OB and differentiating within the granule cell and glomerular layers (Petreanu and Alvarez-Buylla, 2002). The observation that olfactory learning increases the odor-specific survival of adult-born neurons in OB (Alonso et al., 2006; Kermen et al., 2010; Sultan et al., 2010) and, conversely, that the selective activation of these adult-born neurons facilitates olfactory performance and memory (Alonso et al., 2012), has led to a broad and well-supported hypothesis that adult neurogenesis underlies LTM in OB as it does in the hippocampus (reviewed in Sahay et al., 2011; Gheusi et al., 2013; Lepousez et al., 2013). However, the observation that this constant integration of new neurons does not result in a progressively increasing total neuron number in the OB (Mouret et al., 2008) suggests that these new neurons may be relatively short-lived, or may replace older neurons, or both, rendering unclear some essential aspects of the role of adult neurogenesis in long-term odor memory within OB.

In the hippocampus, environmental enrichment and experience increase the survival rates of adult-generated neurons within the dentate gyrus (Kee et al., 2007; Tashiro et al., 2007). Moreover, critically, the selective destruction of adult-born neurons that recently had been incorporated into the hippocampal network impaired spatial memory in the Morris water maze task when animals were tested seven days after training (Arruda-Carvalho et al., 2011). This latter result indicates that these newly-incorporated neurons were substantially mediating the new spatial memory; indeed, it has been suggested that adult-born neurons in HPC are employed specifically for new learning (i.e., initial acquisition), as opposed to the expression or reacquisition of memory (Anderson et al., 2011). A similar principle is emerging in the OB, within which the selective ablation of newly-incorporated adult-born neurons following appetitive odor conditioning eliminated animals' memory for that odor (Akers et al., 2011).

Interestingly, some of the signaling mechanisms most strongly associated with LTM formation also appear to be involved in the learning-dependent survival of adult-born neurons. Besides a basic activity-dependence arising from glutamate and GABA receptor activation (Platel et al., 2010; Platel and Bordey, 2011), the survival of adult-born neurons is also enhanced by stimulation with NE (Veyrac et al., 2009; Moreno et al., 2012) or BDNF (Scharfman et al., 2005). For example, infusions of BDNF into the hippocampus, when delivered to adult rats over 2 weeks, increased the number of adult-born granule cells when compared against control animals infused with saline vehicle or bovine serum albumin (Scharfman et al., 2005). In heterozygous BDNF knockout mice, the number of surviving new neurons in the hippocampus did not change (despite increased proliferation in the SGZ); however, adult-born neurons continued to express markers of immature neurons as well as reduced dendritic growth, suggesting that reduced BDNF levels impaired their processes of maturation and differentiation. Other studies have emphasized a role for BDNF in the survival, rather than the proliferation or differentiation, of adult-born neurons (e.g., Sairanen et al., 2005).

## 4. Mechanisms of odor learning in the OB

Odor learning in the OB offers rare opportunities to study the molecular and structural mechanisms of learning and memory in concert with well-controlled perceptual and behavioral tasks. During appetitive learning, OB circuitry integrates information about the statistical properties of the conditioned stimulus, perhaps also incorporating other features of the odor environment, and supports persistent representations of this learning. Insofar as has been studied, the molecular and structural determinants of OB memory appear similar to those described for hippocampal fear conditioning and other memory systems. The particular value of OB-dependent behavioral learning paradigms is that they enable study of these molecular and structural mechanisms in the more complex milieu of cumulative, multi-trial, representational learning, in which the instantiation of LTM is delayed and conditional in nature, and based on information acquired over time. The representational aspect of OB learning further enables study of how learning alters the form, as well as the strength and persistence, of acquired memories.

### 4.1. Molecular mechanisms in the OB

Intrinsic memory mechanisms within the OB appear to share common pathways and adhere to similar pharmacologically-elaborated phases as have been elucidated in IA-based neural plasticity and memory studies. For example, PKA activity in the neonatal rat OB increases 10 min after one-trial olfactory appetitive conditioning, and blocking PKA activation in the OB with the competitive inhibitor Rp-cAMPS disrupted odor preference memory when tested 5 or 24 h, but not 3 h, after training. Moreover, exogenous administration of the PKA activator Sp-cAMPs into the OB prior to odor exposure sufficed to induce intermediate (5 h) and long-term (24 h) odor preference memory. Higher dosages of Sp-cAMPs into the OB further extended the persistence of this odor preference memory up to 72 h (Grimes et al., 2012). Odor-reward conditioning, but not odor or reward alone, also induced increased CREB phosphorylation in neonatal OB mitral cell nuclei 10 min after training, suggesting that pCREB-related plasticity in mitral cells may be important for the formation of odor LTM (McLean et al., 1999). The MAPK/extracellular signal-related kinase (ERK) pathway also is activated by odor learning in neonates; odor stimulation induced ERK phosphorylation in selective populations of OB neurons related to the identity of the learned odor (Mirich et al., 2004). Neonatal odor learning, like hippocampal LTM, appears to rely on NMDA receptor activation (Lethbridge et al., 2012) and the increased expression of synaptic AMPA receptors (Cui et al., 2011); notably, the PKA-dependent phosphorylation of AMPA receptor subunit GluA1 rises with a similar timecourse as does the level of CREB phosphorylation in mitral cells, peaking at about 10 min post-conditioning (Cui et al., 2011). BDNF mRNA levels increase in the OB and piriform cortex within 2 h of olfactory fear learning (Jones et al., 2007). To the extent that a substantially common set of essential molecular mechanisms is employed, the important distinctions between one-trial learning and appetitive statistical learning become within which neurons, under what conditions, and to what extent these mechanisms are invoked.

Most studies of olfactory learning and memory that measure the form of the odor memory (typically via generalization gradients) have been performed in adult animals and at STM timescales. There is little research to date on the molecular mechanisms underlying bulbar STM, though there is a substantial literature on the effects of neuromodulators, hormones (Dillon et al., 2013), and other extracellular signaling molecules. Noradrenergic effects within OB, in particular, have been studied in both nonassociative and associative olfactory representational learning studies (reviewed in Linster et al., 2011), which suggest that NE in the OB may be necessary for even the simplest forms of odor learning. Notably, a nonspecific infusion of NE into OB suffices to restore the nonassociative learning deficits arising from depletion of cortically-projecting NE fibers (Guérin et al., 2008), though dosage is critical, and bulbar NE levels induced by moderate stress can suppress OB-dependent STM in some contexts (Manella et al., 2013). In neonatal rats, as noted in section 2.2, bulbar NE is heavily released into OB during odor learning, and exogenous application of NE into the OB suffices to induce odor preference learning when paired with an odorant, essentially serving as an unconditioned stimulus as no external source of reward is required (Sullivan et al., 1989; Harley, 2004; Grimes et al., 2012). Activation of the PKA pathway with Sp-cAMPs also acts as an unconditioned stimulus in neonatal OB in this context (Grimes et al., 2012). There is no evidence, however, that bulbar NE can serve as an unconditioned stimulus for adult odor learning, and even in neonates this property may be epiphenomenal. If NE serves to gate activity-dependent plasticity in OB circuits, then known properties of neonatal physiology ensure that in neonates this learning will always be strong, always depend on odor-induced activation of OB circuits, and always be appetitive (neonates respond appetitively and can be positively conditioned to even normally-aversive unconditioned stimuli such as electric shocks; Sullivan, 2001). Consequently, simply gating circuit plasticity in the OB could be expected to directly generate a positive association in neonates. In any event, analogous pairings of odor presentation with bulbar NE infusions in adult mice demonstrate that NE facilitates habituation to presented odors, but does not innately generate odor preferences as it does in neonatal animals (Shea et al., 2008). Of course, other classical neuromodulators, notably acetylcholine acting at muscarinic receptors within OB, also exert effects within OB circuitry on odor learning and STM maintenance (Devore and Linster, 2012; Devore et al., 2012).

BDNF also is clearly implicated in LTM formation for IA learning. While it has been much less thoroughly studied in the olfactory system, BDNF transcription is activated in OB and piriform cortex after odor conditioning (Jones et al., 2007), and olfactory sensory deprivation reduces BDNF expression in neonatal OB (McLean et al., 2001). BDNF and its precursor proBDNF exert distinct physiological effects on OB neuronal excitability and plasticity (Mast and Fadool, 2012). BDNF heterozygous knockout mice and BDNF(Val66Met) point mutants exhibit reduced activity-dependent secretion of BDNF and behavioral deficits in an OB-dependent task. Both mutants habituate normally to odors but exhibit greatly reduced spontaneous discrimination in the cross-habituation task (Bath et al., 2008), suggesting an impairment in their ability to form specific odor representations. The clearest effects of BDNF on the OB, however, are structural in nature, substantially affecting dendritic arborization and adult neurogenesis.

### 4.2. Structural mechanisms in the OB

#### 4.2.1. Long-term potentiation

Long-term potentiation has been clearly if sparsely observed in the early olfactory system, notably within piriform cortex and its ascending synapses into OB. NMDA receptor-dependent LTP has been demonstrated at afferent and associative fiber synapses within piriform cortical slices, and coactivation of the two can facilitate a form of associative LTP if local inhibition is suppressed (Kanter and Haberly, 1990, 1993). Piriform pyramidal neuron feedback projections onto OB granule cells also exhibit spike timing-dependent LTP (Gao and Strowbridge, 2009), which may be a particularly powerful computational element given the importance of dynamical, timing-dependent interactions within OB circuitry. Contemporary models of OB-piriform computations have regarded these circuits as a pattern separation/completion network not unlike the dentate gyrus/CA1 circuit of hippocampus, in which piriform association fibers underlie pattern completion (Hasselmo et al., 1992; Barnes et al., 2008) and their feedback projections onto inhibitory granule cells within OB underlie pattern separation (Strowbridge, 2009), within a common recurrent circuit. This rich and structured plasticity requires further experimental and theoretical development, but exemplifies the capacities of the olfactory system as a model for understanding complex memory systems.

#### 4.2.2. Neuronal and synaptic morphology

Spine densities in OB and piriform cortex are affected by odor learning and by learning-associated trophic factors, notably BDNF. In piriform cortex, spine density on pyramidal neurons increased in odor-conditioned rats compared with pseudoconditioned or naïve controls, an effect potentially corresponding to increased synaptic weights in the association fiber network (Knafo et al., 2001). In the neonatal OB, dendritic branching and spine morphology is substantially regulated by BDNF signaling mediated by the TrkB receptor (Matsutani and Yamamoto, 2004; Imamura and Greer, 2009). In adults, BDNF expression in the OB persists (Malkovska et al., 2006), and continues to regulate OB dendritogenesis, at least among parvalbumin-expressing neurons of the external plexiform layer (Berghuis et al., 2006). In combination with the integration of adult-born neurons into the OB network (below), it is clear that the regulation of dendritic connectivity among OB neurons is a significant determinant of OB functional plasticity, and that BDNF is a crucial regulator of the underlying mechanisms.

#### 4.2.3. Adult neurogenesis

Adult neurogenesis in the OB has been studied extensively with regard to its effects on, and mediation of, odor learning. The differentiation of adult-born neurons within OB and its relevance for olfactory perception and odor learning have been extensively studied and reviewed elsewhere (Lazarini and Lledo, 2011; Lepousez et al., 2013; Gheusi et al., 2013). Of particular interest for present purposes, though, is the regulation of these neuronal differentiation processes by signaling molecules and other established mediators of olfactory learning, as well as timing and task dependencies that may suggest points of particular mechanistic importance.

The incorporation of new neurons is most widely associated with olfactory LTM; as described above, the selective ablation of newly differentiated OB neurons specifically disrupted a long-term odor memory (Akers et al., 2011). However, there also are indications that adult-born neurons may participate in STM processes. Infusions of the antimitotic drug AraC into the lateral ventricle of rats abolished the arrival of new neurons into the OB, while largely sparing hippocampal neurogenesis, and impaired short-term nonassociative memory for odors learned thereafter (Breton-Provencher et al., 2009). Specifically, the absence of new neurons in OB did not affect memory for a habituated odor after 30 min, but 60-, 90-, and 120-min odor memories were disrupted compared with control animals. In contrast, AraC treatment did not affect 24-h or 7-day preference memory for an odorant paired with reward over 4 days. It remains unclear whether this difference depends more on the multi-day spacing of the trials or on the associative nature of the task.

Interestingly, it has been proposed that nonassociative and associative odor learning preferentially activate neurons of different ages within OB (Belnoue et al., 2011). Specifically, nonassociative perceptual learning preferentially activated newly-arrived neurons (~2 weeks old), as measured by c-Fos immunoreactivity, whereas water-rewarded odor discrimination training in a go/no-go task preferentially activated more mature, though still recently generated, interneurons (5–9 weeks of age). This result is consistent with the results described above, in that the OBs of AraC-infused mice in that study were devoid of neurons younger than 3–4 weeks, as required for nonassociative odor learning, but possessed a full complement of neurons in the 5–9 week age range, as were most heavily utilized in the rewarded task. (Also of potential interest is that activation does not necessarily correspond to increased survival; olfactory go/no-go training has been associated with enhancing the survival of 2–4 week old neurons in OB, while increasing apoptosis in 5-week old neurons, and not affecting fully mature interneurons 9 weeks of age or older; Mouret et al., 2008). These results still beg the question, of course, of what factors in these different training paradigms underlie the selective recruitment of different cohorts of new neurons. These results illustrate another advantage of the olfactory system for studies of complex and naturalistic learning, in which task parameters may determine the differential utilization of OB (and non-OB; Luu et al., 2012) circuit elements for odor-dependent learning.

BDNF signaling is a significant contributor to the survival of new neurons in the OB. BDNF levels are similar in both the site of neurogenesis in the SVZ and in the OB, the target of migration, and regulate both neuronal migration and differentiation (the latter via the MAPK pathway) (Petridis and El Maarouf, 2011). Infusions of BDNF into the lateral ventricle of adult rats significantly increased the generation and/or survival of adult-born neurons in the OB (Zigova et al., 1998; Benraiss et al., 2001); in an analogous *in vitro* study, BDNF administered to neurons arising from the subependymal zone of rats promoted their survival (Kirschenbaum and Goldman, 1995). Mice heterozygous for either the BDNF gene or its TrkB receptor exhibit reduced neuron survival in the OB, as do mice with the Val66Met point mutation in the BDNF gene, which impairs activity-dependent BDNF secretion (Bath et al., 2008); these mutants also exhibited impaired nonassociative odor learning as described above. Neuronal proliferation was not affected by these mutations, suggesting that the effects of BDNF primarily relate to survival and differentiation. The powerful effects of this neurotrophin on olfactory learning and neuronal differentiation, and its association with established learning-associated molecular cascades, render it a strong candidate for study in order to elucidate the complex relationships underlying these representational, statistical learning processes in naturalistic contexts.

## 5. In summary

Understanding the neurophysiological basis of natural learning and memory is one of the great challenges of neuroscience. Much of what is known about the cellular mechanisms underlying learning derives from one-trial learning paradigms of inhibitory avoidance (fear conditioning), though research in other plastic neural systems has indicated that they share many, though not all, of the same underlying molecular and structural mechanisms of plasticity. One-trial odor learning studies, which induce plasticity in olfactory bulb, suggest that these cortical circuits also rely on these common mechanisms for plasticity—although bulbar memory also depends on adult neurogenesis, a structural mechanism which it shares only with the hippocampus.

Most natural learning, however, is less categorical than these one-trial paradigms, requiring multiple encounters in order to elucidate relevant stimuli and learn appropriate associations. Appetitive learning in adults, for example, tends to be gradual, conditional, and statistical in nature. This raises new mechanistic questions: how does learning accumulate over multiple trials? How do STM and LTM mechanisms interact over the extended timescales of natural experience? How are the relevant features of the sensory scene identified, selected, and represented? How does learning change the form, or quality, of a sensory representation in response to accumulating information? Developing the olfactory system as a neurophysiological learning and memory model enables engagement with these rich questions.

## Author contributions

Michelle T. Tong and Thomas A. Cleland conceived of and wrote the paper, and designed the figures. Shane T. Peace designed and performed the research featured in Figure 4. Michelle T. Tong designed and performed the research featured in Figure 5.

## Funding

This work was supported by NIH/NIDCD grant DC012249 to Thomas A. Cleland.

### Conflict of interest statement

The authors declare that the research was conducted in the absence of any commercial or financial relationships that could be construed as a potential conflict of interest.
